# The effect of rocking speed and cell inoculation density on hepatic spheroid size: A simple method for size modulation

**DOI:** 10.1002/btm2.70154

**Published:** 2026-06-05

**Authors:** Michael G. Megaly, Diane Tobolt, Anthony An‐Fa Dahm Chen, Bat‐Erdene Namsrai, Benjamin Fisher, Joseph Sushil Rao, Srivasupradha Ramesh, Anudari Chimedtseren, Scott L. Nyberg, Mark Clemens, Charles Y. Lee, John C. Bischof, Erik B. Finger

**Affiliations:** ^1^ Division of Transplantation, Department of Surgery University of Minnesota Minneapolis Minnesota USA; ^2^ Department of Mechanical Engineering University of Minnesota Minneapolis Minnesota USA; ^3^ Schulze Diabetes Institute University of Minnesota Minneapolis Minnesota USA; ^4^ William J von Liebig Transplant Center Mayo Clinic College of Medicine Rochester Minnesota USA; ^5^ Department of Biological Sciences University of North Carolina Charlotte North Carolina USA; ^6^ Department of Mechanical Engineering and Engineering Science University of North Carolina Charlotte North Carolina USA; ^7^ Institute for Engineering in Medicine University of Minnesota Minneapolis Minnesota USA

**Keywords:** cell density, hepatocyte spheroids, organoid, rocker speed, spheroid size

## Abstract

Hepatic spheroids are widely used in bioartificial liver (BAL) systems, hepatotoxicology, and pharmacology due to improved functional stability compared with monolayer cultures. However, many existing methods rely on specialized equipment or static platforms that limit scalability and dynamic culture applications. Primary rat hepatocytes were cultured as three‐dimensional spheroids using a rocker‐based suspension system. Spheroid formation was evaluated across a range of rocking speeds (7–18 cycles per minute [cpm]) and inoculation densities. Spheroid morphology was quantified over time using two‐dimensional imaging. Functional performance was assessed by urea and albumin production on days 1, 4, 7, and 10. Spheroid formation efficiency was evaluated using volumetric modeling. Mean spheroid diameter decreased from 199 ± 41 μm at 7 cpm to 80.2 ± 17.6 μm at 18 cpm. The spheroid area was significantly influenced by both culture day (*p* < 0.0001) and rocker speed, with significant differences observed across speeds at all time points (*p* < 0.001, days 1–7). Mean spheroid area at 10 cpm was 13,750 ± 9051 μm^2^ (day 1), 26,730 ± 8658 μm^2^ (day 4), and decreased to 22,526 ± 8705 μm^2^ (day 7). Urea production differed across speeds (ANOVA, *p* = 0.043), with peak performance at 12 cpm (median 3.47 μg/μg DNA) compared to 7 cpm (median 0.11 μg/μg DNA). Albumin production was 1086.8 ng/μg DNA on day 1 and 1534.4 ng/μg DNA on day 10. Urea production was 0.62 μg/μg DNA on day 1 and 1.25 μg/μg DNA on day 10. Median hepatocyte incorporation was 86% (range 73%–116%) by day 4. This rocker‐based method provides a simple, low‐cost, and scalable approach for generating hepatic spheroids with tunable size distributions under dynamic conditions. The system supports progressive functional maturation and is compatible with established scale‐up strategies, supporting its potential for translational applications.

## Graphical abstract

This visual abstract demonstrates that rat hepatic spheroid size can be predictably controlled by adjusting rocker speed and cell inoculation density in a dynamic culture system. Increasing speed produces smaller, more uniform spheroids, while higher density increases size. Intermediate speeds optimize function, supporting scalable, low‐cost spheroid production for translational applications.

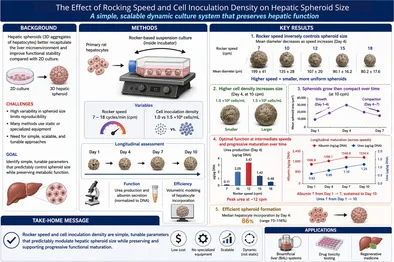

**Translational Impact Statement**

Hepatic spheroids are increasingly used in bioartificial liver systems, drug toxicology testing, and regenerative medicine, yet variability in spheroid size limits reproducibility and scale‐up. This study identifies rocker speed and cell inoculation density as simple, tunable parameters that enable predictable control of spheroid size while preserving metabolic function. These findings provide a practical framework for standardizing hepatic spheroid production and accelerating translation of hepatocyte‐based technologies.

**Abbreviations**
cpm: cycles per minute
SCM: spheroid culture media
SFM: spheroid forming media

## 1 INTRODUCTION

Liver‐derived cellular systems, including isolated hepatocytes, organoids, tissue slices, and whole organ models, are increasingly sought after for diverse applications such as bioartificial liver (BAL) systems, hepatotoxicity testing, and regenerative medicine. To use BAL as an example, transplantation remains the gold standard for treating end‐stage liver failure; however, organ availability lags far behind demand; therefore alternatives are crucial. Unlike end‐stage renal disease, liver failure lacks a widely available, durable extracorporeal bridging therapy comparable to dialysis. BALs, which incorporate functional hepatocytes to replicate liver function, offer a promising alternative. The widespread clinical application is constrained by the limited availability and viability of functional hepatocytes.^1^, ^2^, ^3^

Hepatic spheroids are three‐dimensional aggregates of hepatocytes (organoids) that better recapitulate the in vivo liver microenvironment than monolayer cultures and have therefore transformed experimental hepatology by enabling more physiologically relevant liver models. These spheroids exhibit preserved cellular polarity, intracellular communication, and liver‐specific functions (e.g., albumin and urea production) as well as demonstrate improved longevity and phenotypic stability compared with two‐dimensional cultures.^3^, ^4^, ^5^, ^6^ Their attributes have translated to enhanced function, survival, and resistance to cryopreservation‐associated damage, as extensively reviewed in literature.^7^ Spheroid size is critical for the health of the spheroid, as it influences oxygen diffusion, nutrient transport, and core viability. Larger aggregates are at increased risk of developing hypoxic or necrotic cores.^8^, ^9^, ^10^ Since spheroids are a good representation of the in vivo liver microenvironment, they are of great importance for the advancement of liver science and medicine in allowing for a vast array of experimentation with substantially less liver mass. Therefore, control of spheroid size is essential for reproducible and scalable hepatic spheroid formation.

There are many spheroid formation methods including galactose density, micro‐molding, hanging drop culture, ultra‐low attachment plates, spinner culture, medium component, external forces, coculture system, and polymeric nanospheres to name a few.^11^, ^12^ While all are viable options, the majority require special instrumentation, which could be cost prohibitive for some labs. The spinner flask method is a low‐cost method; however, the spheroids can experience a strong shearing force, which is less than ideal when culturing stressed spheroids involved in experimentation. Ultra‐low attachment (ULA) plate systems are widely used for spheroid generation and can produce highly uniform spheroids of low variability with coefficients of variation in the range of 10%,^13^ however these require a static environment for production, the plates themselves, and are more difficult to scale when needed. The rocker method allows for a tunable and scalable approach for generating hepatic spheroids with predictable size distributions that is simple to implement and low‐cost. Therefore, we employed the rocker method to form hepatic spheroids.^14^, ^15^, ^16^, ^17^ We hypothesized that rocker speed and inoculation density represent simple, readily adjustable parameters that directly influence spheroid size in a predictable manner. Accordingly, the objective of this focused investigation was to determine how modulation of these variables could be used to produce spheroids of predictable and tunable dimensions, thereby improving reproducibility and facilitating downstream applications such as BAL, hepatotoxicology testing, and biomedical research.

## 2 MATERIALS AND METHODS

### 2.1 Study design: hepatocyte isolation

Sprague–Dawley rats (8–12‐week‐old males, weighing 200–250 g, Charles River Labs) were used, with hepatocytes from one rat per isolation supplying each experimental condition. Animals were fasted overnight, anesthetized, and heparinized. Rats were euthanized via exsanguination while under general anesthesia using 1%–4% Isoflurane in oxygen. Livers were surgically procured and flushed with 30 mL of University of Wisconsin (UW) solution. Organs were perfused ex vivo using a normothermic perfusion system with a 1× Krebs–Henseleit Buffer priming solution for 10 min, followed by 10 min of collagenase perfusion. Hepatocytes were isolated via gentle mechanical disruption of the liver capsule, filtered, and washed. Cell viability was assessed with trypan blue exclusion. Viability ranged from 95% to 98%. The University of Minnesota Institutional Animal Care and Use Committee (IACUC) approved this protocol (protocol #2504‐42888A). Animals were housed in individually ventilated cages (IVC, 2 rats per cage) under controlled temperature (22 ± 2°C) and humidity (55% ± 10%), with a 12‐h light/dark cycle (lights on at 07:00). Standard chow and water were provided ad libitum. Environmental enrichment included nesting material, cardboard tunnels, and wooden blocks. All husbandry was performed according to institutional animal care guidelines. Each experimental condition was derived from a single hepatocyte isolation to minimize inter‐isolation variability and to focus specifically on the effects of mechanical and seeding parameters on spheroid morphology.

### 2.2 Spheroid formation

For this study, we generated spheroids from rat hepatocytes using the rocker method.^16^ Hepatocytes were plated at either 1 × 10^6^ or 1.5 × 10^6^ cells/mL in 20 mL spheroid formation media (SFM) consisting of a supplemented Williams E medium into rectangular Mayo dishes (10 cm x 8 cm x 2 cm). To evaluate for potential instrument‐dependent variability, dishes were placed onto one of two distinct rocker platforms (Hi‐Lo Rocker, model ROCAA115S (IBI Scientific, Dubuque, IA, USA)) or standard Bellco Biotechnology Rocker Base Unit, 100 V SKU: 7740‐01000 (Bellco Biotechnology, Vineland, NJ, USA) under matched conditions of rocking speed, tilt angle (7°–11°), and inoculation density. Comparable spheroid size distributions were observed across platforms following parameter optimization. Rocker platforms were housed inside a Thermo Scientific Napco Series 8000 DH Water‐Jacketed CO_2_ Incubator (Thermo Fisher Scientific, Waltham, MA, USA) (37°C, 5% CO_2_, 95% humidity) (Figure 1). Rocker speeds tested were 7, 10, 12, 15, and 18 cpm each across days 1–7. After 24 h, SFM was exchanged for spheroid culture media (SCM).^16^, ^18^ This process generated homogeneous spherical cell aggregates with smooth borders and nearly 100% viability by acridine orange (green)/propidium iodide (red) live dead cell stain (Figure 2). Morphological dynamics varied with both culture day and rocker speed, as evidenced by differences in spheroid area, perimeter, and major and minor axis length (Figure 3a).

**FIGURE 1:**
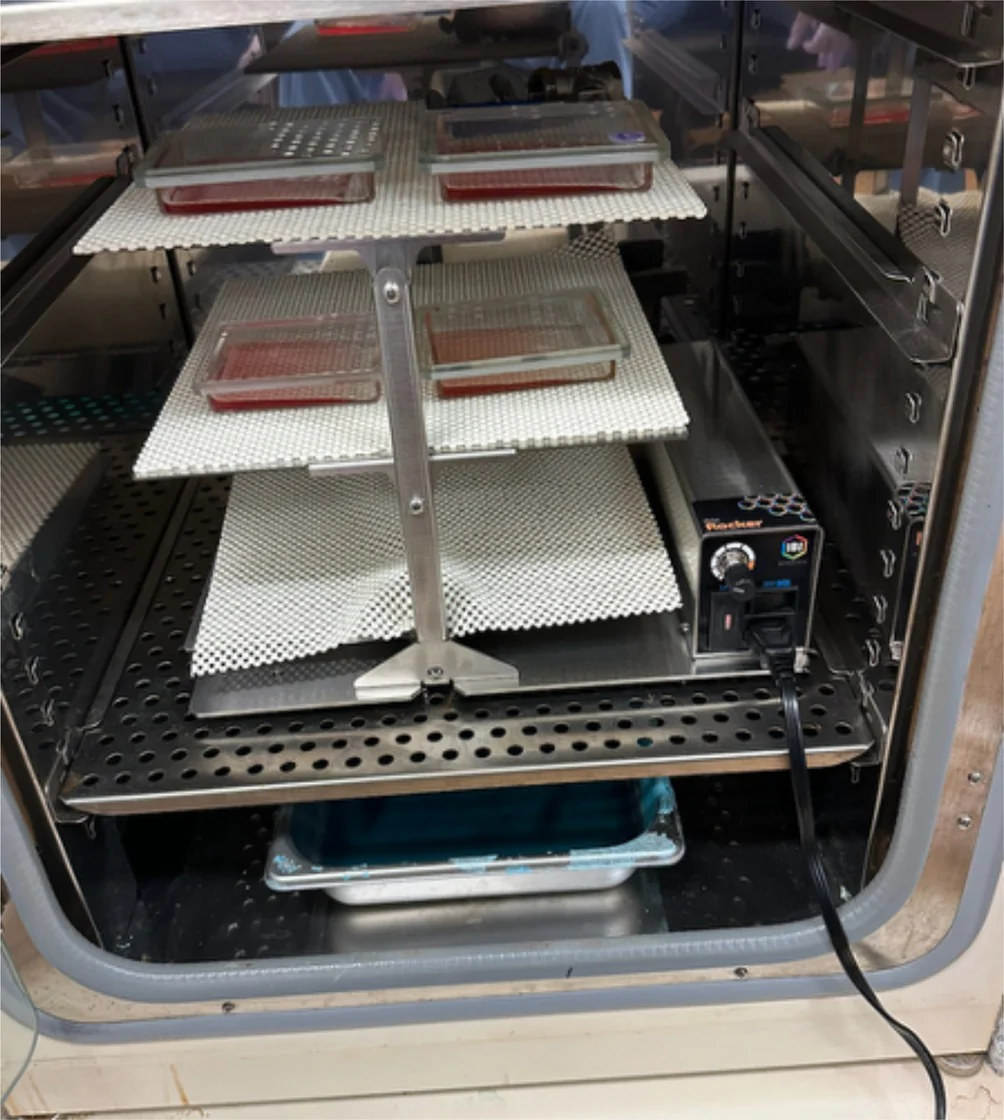
Experimental setup showing hepatocyte plating and rocker platform. Hepatocyte spheroids are formed in a tissue culture incubator while under constant rocking motion on Hi‐Lo Rocker, model ROCAA115S (IBI Scientific, Dubuque, IA, USA).

**FIGURE 2:**
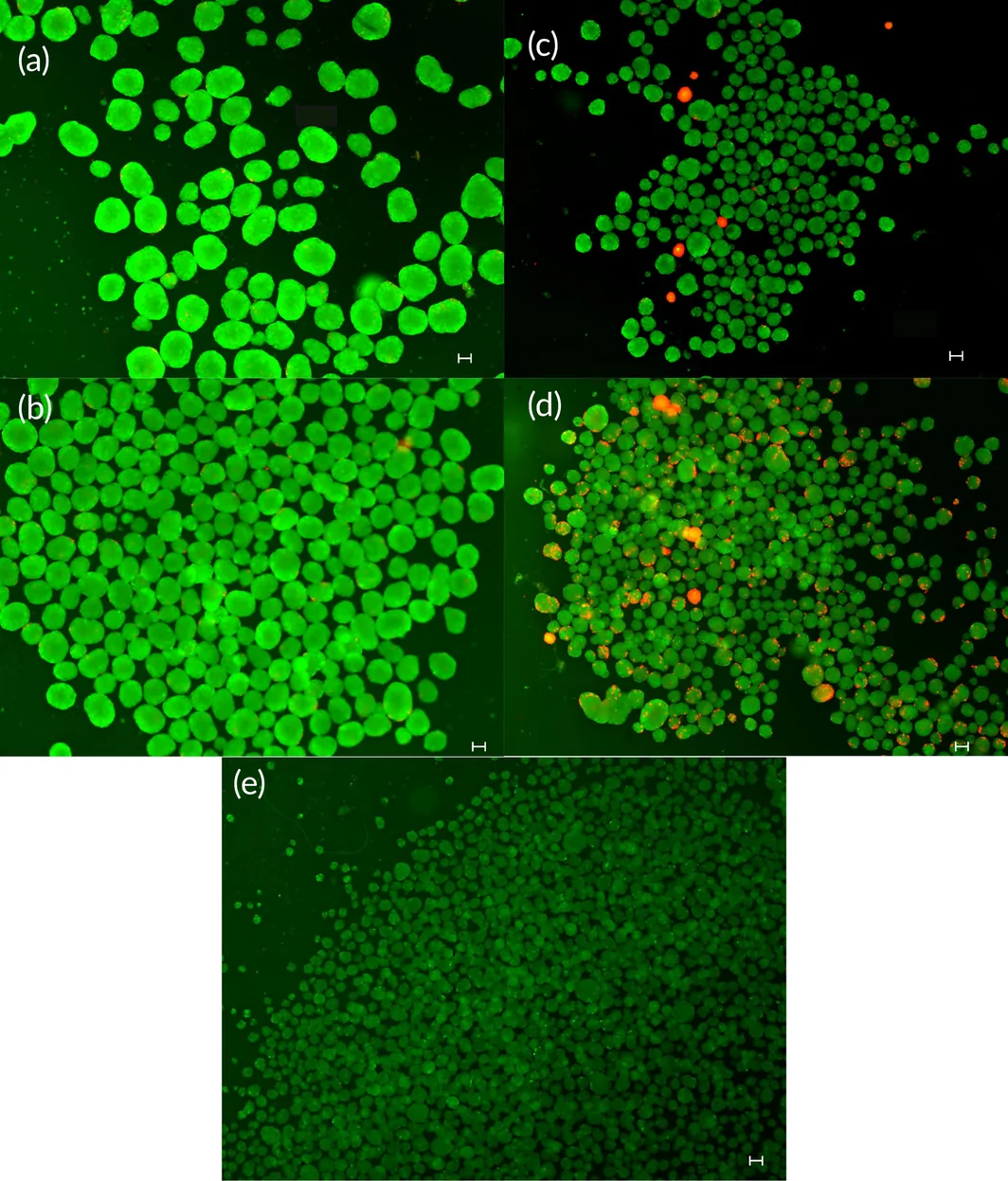
Viability, morphology, and size of hepatocyte spheroids formed at different rocking speeds. Representative images of spheroids formed at different rocker speeds (7–18 cpm) under fluorescent microscopy using acridine orange (green)/propidium iodide (red) viability stain at day 3 post hepatocyte isolation. (a) Rocker speed 7 cpm, (b) 10 cpm, (c) 12 cpm, (d) 15 cpm, (e) 18 cpm. Scale bar = 100 μm. cpm, cycles per minute.

**FIGURE 3:**
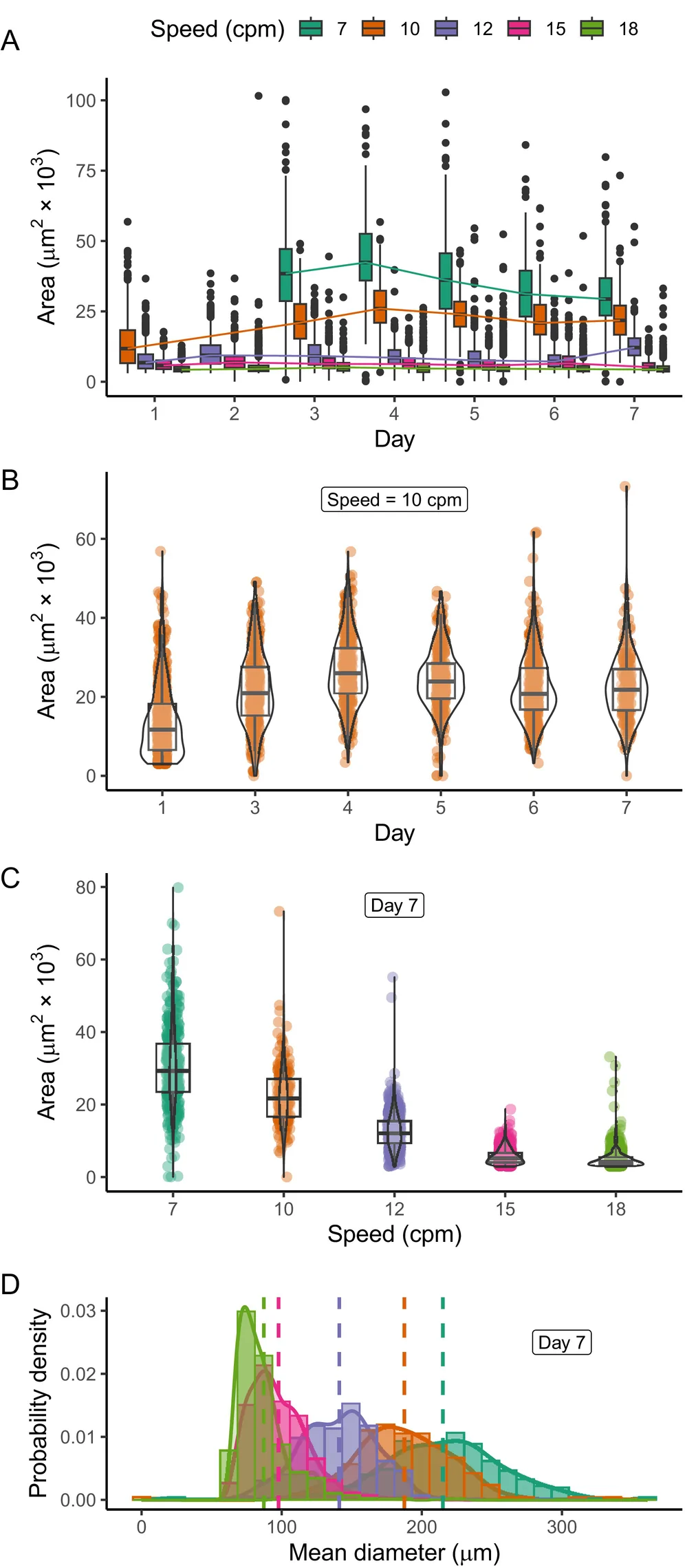
Hepatocyte spheroid area increases with decreasing rocking speed. (a) Spheroid area at different rocker speeds over the course of 7 days. (b) Comparison of spheroid area over 7 days at speed 10 cpm. (c) Comparison of spheroid area at speeds 7–18 cpm at day 7. (d) Mean diameter of spheroids at speeds 7–18 cpm at day 7. Vertical lines represent the mean for each speed. Data are box plots (± superimposed violin plot) or histogram. cpm, cycles per minute.

#### 2.2.1 Spheroid formation efficiency and volumetric analysis

Spheroid formation efficiency was evaluated under a focused assessment representative rocker condition of 9 cycles per minute (cpm). Spheroids were allowed to form under aforementioned rocker culture conditions and were imaged on day 4. The spheroid size was quantified with a Keyence BZ‐X series microscope, with measurement of spheroid area and major and minor axes. Spheroid volume was estimated assuming an ellipsoidal geometry. Total spheroid volume per sample was calculated as the sum of individual spheroid volumes.

Hepatocyte volume was estimated assuming a spherical geometry with a diameter of 17.5 μm.^19^, ^20^ Total initial hepatocyte volume was calculated by multiplying per‐cell volume by the total number of seeded cells. Spheroid formation efficiency was defined as the percentage of initial hepatocyte volume incorporated into spheroid structures, calculated as:

$$\text{Incorporation}\;\left(\%\right)=\left(\text{total spheroid volume}/\text{initial hepatocyte volume}\right)\times 100$$

This analysis was performed across five independent datasets at day 4.

### 2.3 Urea and albumin measurements

Urea production was evaluated using the QuantiChrom Urea Assay Kit (QuantiChrom™ Urea Assay Kit, DIUR‐500; BioAssay Systems, Hayward, CA). One milliliter of spheroid suspension was washed with phosphate‐buffered saline (PBS) and then incubated for 2 h in arginase‐free Krebs–Henseleit–Hepes (KHB) buffer as described by Peters et al.^21^ Urea was subsequently normalized to DNA. DNA content was measured using the Quant‐iT PicoGreen dsDNA Assay Kit (Invitrogen, Thermo Fisher Scientific; Cat. P11496) according to the manufacturer's instructions. Urea concentrations were measured over the course 4 distinct days: days 1, 4, 7, 10 post isolation.

Albumin was measured using a quantitative enzyme‐linked immunosorbent assay (ELISA) (Rat Albumin ELISA Kit, Catalog No. E‐25AL, Immunology Consultants Laboratory, Inc., Portland, OR, USA). Spheroids were cultured for 24 h. Albumin concentrations were determined according to the manufacturer's instructions using a standard curve generated from known albumin concentrations. Absorbance was measured at 450 nm using a microplate reader (Synergy 2, BioTek Instruments, Winooski, VT, USA). Albumin was normalized to spheroid DNA content similarly to urea. Results were expressed as albumin per μg DNA over the collection interval (e.g., ng/μg DNA). Albumin concentrations were measured over the course 4 distinct days: days 1, 4, 7, 10 post isolation.

For urea and albumin, each condition was assessed in biological and technical replicates (≥3 wells per condition) across at least three independent experiments each at 9 cpm.

### 2.4 Imaging and analysis

Spheroids were imaged daily for 7 days using a Keyence BZ‐X810 inverted all‐in‐one fluorescence microscope (Keyence Corporation of America, Itasca, IL, USA) (Figures 2 and 3). Using the Keyence analysis tool, major and minor axes and spheroid area were measured, and volume was calculated from these parameters. Acridine Orange (5 μg/mL) and Propidium Iodide (10 μg/mL) live‐dead cell stains were used to evaluate the spheroids under fluorescence microscopy. Each experiment corresponded to a different rocker speed, with thousands of spheroids analyzed per condition across multiple dishes.

### 2.5 Statistical analysis

Statistical analysis was performed in R v. 4.5.0 (R Foundation for Statistical Computing, Vienna, Austria). The primary outcome measures were spheroid sized parameters including area, perimeter, long axis diameter, and short axis diameter. Spheroid sizes were reported as medians and interquartile ranges (IQR) or mean ± SD, except where specifically noted otherwise. Samples were tested for normality using the Shapiro–Wilk test and for homogeneity of variance using Levene's test. Group differences were analyzed using the Kruskal–Wallis test with post hoc Mann–Whitney *U* comparisons. A *p* value <0.05 was considered statistically significant. To quantify variability in spheroid size distributions, dispersion was assessed using the coefficient of quartile variation (CQV). For each condition, spheroid size data were summarized by the first (Q1) and third (Q3) quartiles, and CQV was calculated as (Q3 − Q1)/(Q3 + Q1).

## 3 RESULTS

### 3.1 Effect of rocking speed

#### 3.1.1 Within‐speed trends

Compact and moderately uniform cell aggregates (spheroids) formed within 1 day for all speeds except 7 cpm, where consistent spheroids were not observed until day 3. Apart from 7 cpm, the spheroid sizes showed consistent growth patterns. From days 1 to 4, the spheroid areas increased, then decreased between days 4 and 5. By way of example, at a speed of 10 cpm, spheroids were observed from day 1, with a mean area of 13,750 ± 9051 μm^2^ (Figure 3b, Table 1). The size increased to 21,807 ± 9247 μm^2^ by day 3 and peaked at 26,730 ± 8658 μm^2^ on day 4. After hitting this peak, the size began to contract, measuring 24,357 ± 7689 μm^2^ on day 5, 22,283 ± 8435 μm^2^ on day 6, and 22,526 ± 8705 μm^2^ on day 7. Similar patterns of size changes were observed at speeds of 12, 15, and 18 cpm.

**TABLE 1:** Summary of size parameters over days 1–7 (at speed = 10 cycles per minute).

Variable	*N*	1, *N* = 551^a^	3, *N* = 364^a^	4, *N* = 345^a^	5, *N* = 305^a^	6, *N* = 361^a^	7, *N* = 168^a^	*p*‐value^b^
Minor axis (μm)	2094	114.0 (83.0, 142.0)	155.0 (132.5, 176.0)	174.0 (154.0, 192.0)	167.0 (151.0, 182.0)	155.0 (139.0, 177.0)	157.5 (138.5, 176.0)	<0.001
Major axis (μm)	2094	141.0 (104.0, 176.0)	183.5 (156.5, 209.0)	198.0 (176.0, 222.0)	192.0 (174.0, 213.0)	181.0 (161.0, 205.0)	186.0 (164.0, 210.0)	<0.001
Perimeter (μm)	2094	416.0 (307.0, 523.0)	552.0 (469.5, 630.5)	606.0 (545.0, 680.0)	587.0 (529.0, 643.0)	543.0 (487.0, 620.0)	566.5 (497.0, 624.5)	<0.001
Area (×10^3^ μm^2^)	2094	11.8 (6.6, 18.4)	21.0 (15.3, 27.6)	26.0 (20.9, 32.4)	23.9 (19.6, 28.5)	20.9 (16.8, 27.3)	21.8 (16.7, 27.1)	<0.001
Volume (×10^6^ μm^3^)	2094	0.9 (0.4, 1.8)	2.2 (1.4, 3.3)	3.0 (2.2, 4.1)	2.7 (2.0, 3.5)	2.1 (1.6, 3.3)	2.3 (1.6, 3.2)	<0.001

^a^ Median (Q1, Q3).

^b^ Kruskal–Wallis rank sum test.

#### 3.1.2 Between‐speed comparisons

Nonparametric analysis using the Kruskal–Wallis test demonstrated significant effects of culture day (*p* < 0.0001) and a significant rocker speed × day interaction (*p* < 0.0001) on spheroid area, indicating that spheroid size evolves over time in a speed‐dependent manner. Across all measured time points, increasing rocker speed was associated with progressively smaller spheroid areas.

Day‐by‐day comparisons confirmed significant differences in spheroid area across rocker speeds at every culture day (*p* < 0.001 for days 1–7). Post hoc Mann–Whitney analyses demonstrated a consistent inverse relationship between rocker speed and spheroid size, with lower speeds (7–10 cpm) producing significantly larger spheroids and higher speeds (15–18 cpm) yielding significantly smaller aggregates, particularly from days 3 through 7 (Table 2).

**TABLE 2:** Summary of size parameters over different forming speeds (at day 7).

Variable	*N*	7, *N* = 362^a^	10, *N* = 168^a^	12, *N* = 531^a^	15, *N* = 926^a^	18, *N* = 688^a^	*p*‐value^b^
Minor axis (μm)	2675	183.5 (165.0, 205.0)	157.5 (138.5, 176.0)	118.0 (103.0, 133.0)	77.0 (68.0, 88.0)	70.0 (63.0, 79.0)	<0.001
Major axis (μm)	2675	216.0 (187.0, 242.0)	186.0 (164.0, 210.0)	142.0 (121.0, 160.0)	88.0 (78.0, 102.0)	82.0 (73.0, 93.0)	<0.001
Perimeter (μm)	2675	657.0 (579.0, 730.0)	566.5 (497.0, 624.5)	429.0 (366.0, 479.0)	268.0 (235.0, 305.0)	245.0 (221.0, 280.5)	<0.001
Area (×10^3^ μm^2^)	2675	29.4 (23.5, 36.8)	21.8 (16.7, 27.1)	12.1 (9.3, 15.5)	5.2 (4.1, 6.7)	4.3 (3.6, 5.5)	<0.001
Volume (×10^6^ μm^3^)	2675	3.6 (2.6, 5.2)	2.3 (1.6, 3.2)	1.0 (0.7, 1.4)	0.3 (0.2, 0.4)	0.2 (0.1, 0.3)	<0.001

^a^ Median (Q1, Q3).

^b^ Kruskal–Wallis rank sum test.

Assessment of variance using Levene's test revealed significant heterogeneity in spheroid size distributions across speeds at all time points (*p* < 0.0001 at days 1–7). Size variability was greatest at the slower speeds, most notably at 7 cpm, which exhibited the widest interquartile ranges. Mean spheroid diameters (average of long and short axis) decreased with increasing rocker speed, measuring 199 ± 41.2 μm at 7 cpm, 173 ± 33.4 μm at 10 cpm, 129 ± 25.4 μm at 12 cpm, 90.9 ± 18.4 μm at 15 cpm, and 80.2 ± 17.6 μm at 18 cpm (Figure 3d). The width of distribution was greatest at the slowest speed and largest spheroid diameter (speed 7 cpm), which correspondingly had the largest standard deviation.

To further characterize spheroid size heterogeneity, dispersion was assessed using the coefficient of quartile variation (CQV). CQV values generally ranged from 18% to 37%, varying as a function of rocking speed and time in culture. At earlier time points, CQV values were higher (up to 47% on one occasion for day 1, 7 cpm), reflecting increased heterogeneity during initial aggregation. Over time, dispersion decreased and stabilized, with CQV values converging to 21%–24% by day 7 across all speeds. Notably, higher rocking speeds were generally associated with lower CQV values compared to slower speeds, indicating a consistent relationship between dynamic culture conditions and spheroid size distribution (Figure S1).

### 3.2 Effect of cell density

Spheroid growth kinetics were compared between inoculation densities of 1.0 × 10^6^ cells/mL and 1.5 × 10^6^ cells/mL, with both conditions cultured at 15 cpm (Figure 4). At 1.0 × 10^6^ cells/mL, mean spheroid area measured 6273 ± 2402 μm^2^ on day 1, increased modestly by day 2 (7271 ± 3378 μm^2^), and subsequently stabilized, measuring 6879 ± 2840 μm^2^ on day 4 and 5627 ± 2021 μm^2^ on day 7.

**FIGURE 4:**
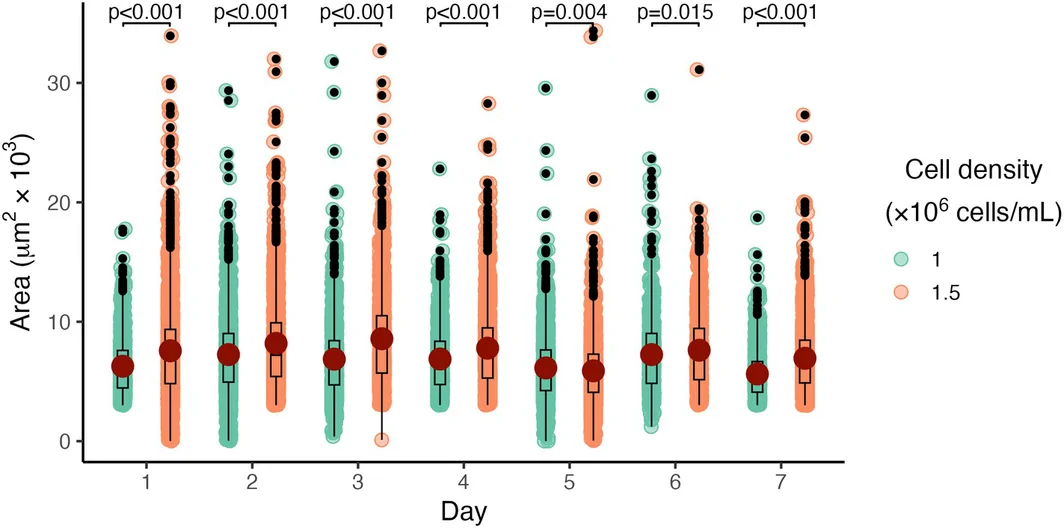
Comparison of hepatocyte spheroid size based on inoculation density over 7 days. Spheroids were formed at 15 cpm with hepatocyte inoculation densities of 1 M/mL and 1.5 M/mL. Data are box and whisker plots. *p* values are from the Mann–Whitney *U* test.

In contrast, spheroids formed at 1.5 × 10^6^ cells/mL were consistently larger at all time points. Mean spheroid area was 7655 ± 4951 μm^2^ on day 1, increased to 8249 ± 4271 μm^2^ on day 2 and 9712 ± 4532 μm^2^ on day 3, and remained larger than the lower‐density condition by day 7 (7203 ± 3146 μm^2^ vs. 5627 ± 2021 μm^2^).

Across all culture days, higher inoculation density was associated with significantly increased spheroid area (*p* < 0.05 for all days, Mann–Whitney *U* test). A significant interaction between inoculation density and culture day (*p* < 0.001) further indicated that the magnitude of this effect varied over time. Collectively, these findings demonstrate that increasing inoculation density from 1.0 × 10^6^ to 1.5 × 10^6^ cells/mL produces significantly larger spheroids throughout the 7‐day culture period, while preserving the overall temporal pattern of early expansion followed by size stabilization (Figure 4).

### 3.3 Spheroid formation efficiency

Spheroid formation efficiency increased over time, with early time points demonstrating incomplete incorporation and later time points reflecting progressive cellular aggregation.

Estimated incorporation values were 95%, 73%, 86%, 77%, and 116% for Datasets 1–5, respectively (Figure S2). The median incorporation was 86%, with a range of 73%–116%. Four of five datasets demonstrated incorporation below 100%. Across datasets, incorporation values consistently clustered between approximately 70% and 95%, supporting efficient spheroid formation by day 4.

### 3.4 Effect of rocker speed on hepatocyte function‐ urea and albumin production

Urea production normalized to DNA varied across rocker speeds, demonstrating a speed‐dependent effect on hepatocyte metabolic function. At 7 cpm, urea production was minimal, with values clustering near zero (median ~ 0.11 μg/μg), spheroids cultured at 9 cpm exhibited a marked increase in urea production (median ~ 1.68 μg/μg), at 12 cpm a median value of approximately 3.47 μg/μg and a relatively tight interquartile range. At higher speeds, urea production declined. At 15 cpm, median urea production decreased to ~1.72 μg/μg with increased variability, including both high and low outliers. Similarly, at 18 cpm, urea production remained lower (median ~ 1.02 μg/μg). Killed controls demonstrated negligible urea production (median approximately −0.08 μg/μg), confirming assay specificity and appropriate normalization. (Figure 5). There is a statistically significant difference between urea production at 7 cpm relative to 9 and 12 cpm (*p* value <0.05). However there is no statistically significant difference between urea production for speed 9, 12, 15, and 18.

**FIGURE 5:**
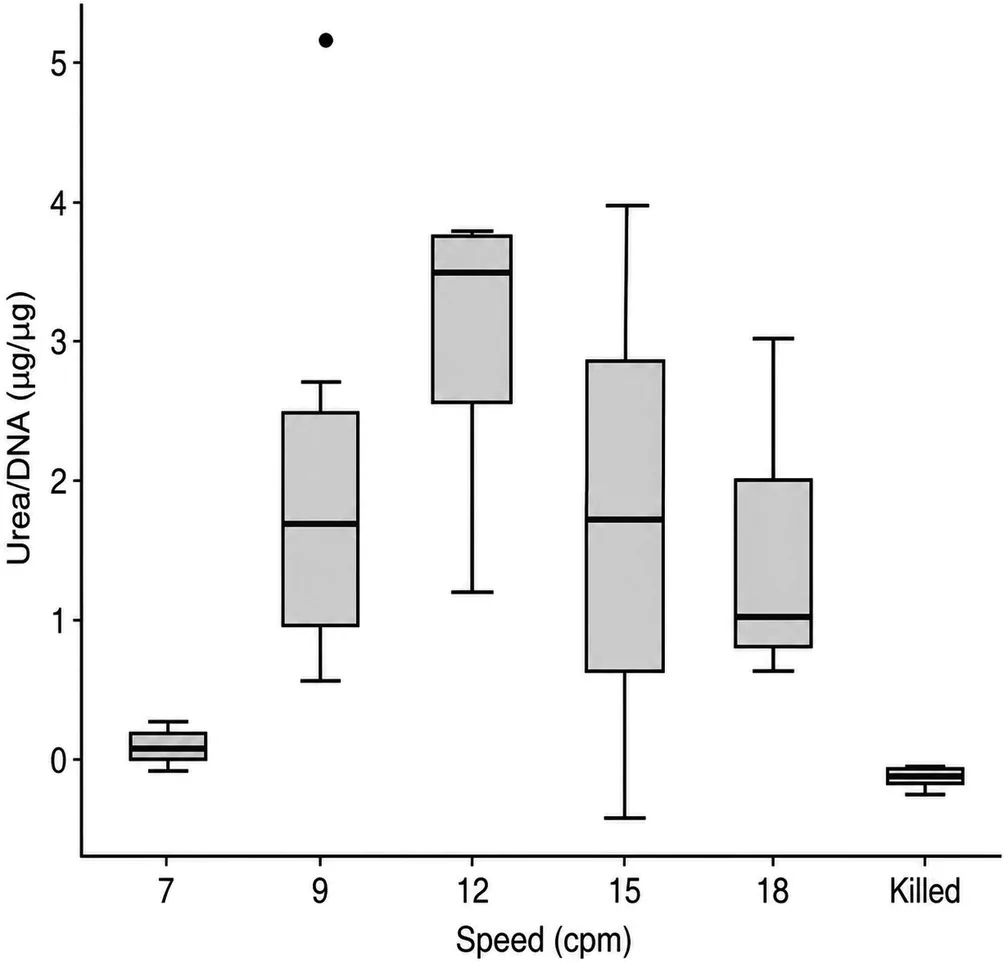
Urea production normalized to DNA content across rocker speeds. Hepatocyte spheroids were cultured at varying rocker speeds, and metabolic function was assessed by measuring urea production normalized to total DNA content. Individual data points represent biological replicates, with mean ± standard deviation overlaid. Killed hepatocytes are shown as a negative control. Intermediate rocking speeds (9–15 cpm) demonstrate higher urea/DNA production compared with very low (7 cpm) and high (18 cpm) speeds. All experiments are triplicate at day 5.

Both albumin production and urea synthesis increased over time. (Figure 6) Samples were taken from cultures at 9 cpm. Albumin production showed a gradual increase from days 1 to 10. At day 1, median albumin production was 1086.8 ng/μg DNA (IQR 976.2–1528.2). By day 4, albumin production increased modestly to a median of 1122.0 ng/μg DNA (IQR 782.5–1753.5), with substantial variability observed across samples. A more pronounced increase was observed at day 7, with a median of 1545.8 ng/μg DNA (IQR 1174.1–1803.5). By day 10, albumin production remained elevated with a median of 1534.4 ng/μg DNA (IQR 1442.6–1962.8), and higher upper‐range values (up to ~2385 ng/μg DNA).

**FIGURE 6:**
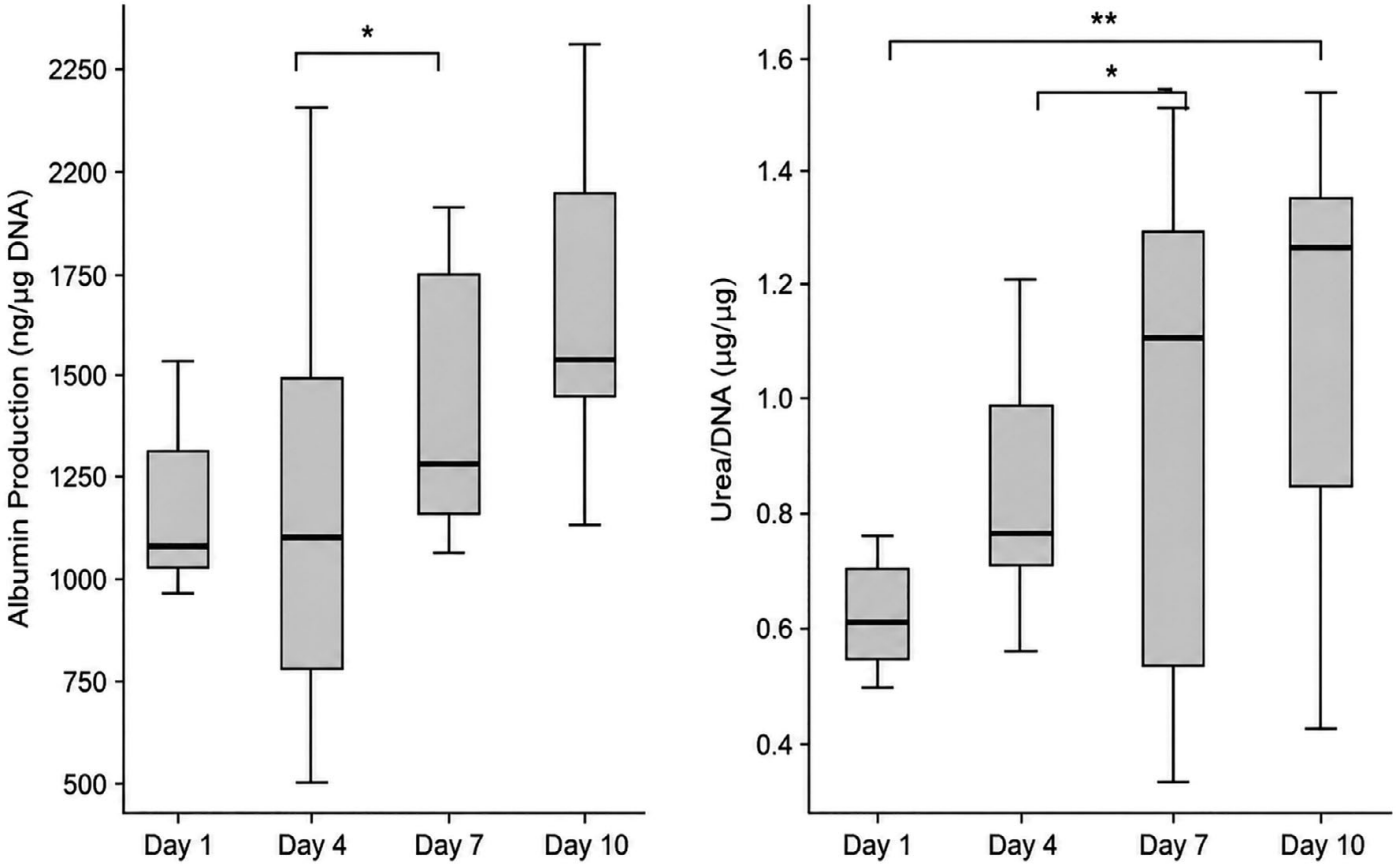
Albumin production (left) and urea production normalized to DNA (right) in hepatocyte spheroids cultured at 9 cpm over time. Data are presented as median with interquartile range. Albumin production (per 24 h) demonstrated a significant increase between days 4 and 7 (*p value <0.05). Urea production (per 2 h) similarly increased over time, with a significant increase observed between days 4 and 7 (*p < 0.05) and between days 0 and 10 (**p < 0.01), consistent with progressive hepatocyte functional maturation followed by stabilization.

Urea production normalized to DNA similarly increased over time, with a more marked relative change compared to albumin. Median Urea/DNA increased from 0.6209 μg/μg (IQR 0.523–0.731) at day 1 to 0.7761 μg/μg (IQR 0.697–1.066) at day 4, followed by a substantial increase to 1.0996 μg/μg (IQR 0.691–1.379) at day 7. By day 10, urea production further increased to a median of 1.2538 μg/μg (IQR 0.845–1.398). Both functional markers demonstrated a similar temporal pattern, with relatively modest changes between Days 1 and 4 followed by a more pronounced increase between Days 4 and 7.

## 4 DISCUSSION

This study demonstrates a simple relationship between rocker speed, cell density, and spheroid size. As hypothesized, the faster rocking speeds result in smaller spheroids, likely due to increased shear forces and reduced contact time for hepatocyte aggregation.^22^, ^23^ Conversely, slower speeds allow for greater cell–cell interaction and larger spheroid formation. However, below a threshold (i.e., 7 cpm), spheroid formation becomes inconsistent, possibly due to insufficient kinetic energy for aggregation.^23^ At a speed of 7 cpm, consistent spheroids were not formed until day 3 in half of the Mayo dishes, while some Mayo dishes did not form spheroids at all. The aim of this study is to evaluate how modulation of rocker speed and inoculation density could be used to produce spheroids of predictable and tunable dimensions, thereby improving reproducibility and facilitating downstream applications. We explicitly make no attempt to define a single optimal speed or time point for the optimal use of spheroids, as these are parameters that are likely dependent on the hepatocyte source and experimental context.

When keeping speed constant, increased inoculation density also yielded larger and more numerous spheroids, likely due to a greater probability of cell–cell interaction and aggregate stability.^24^, ^25^ These findings are particularly relevant for applications requiring controlled spheroid size and diffusion geometry, including hepatotoxicology and BAL seeding strategies.^2^, ^7^

Shear stress from faster rocking may interfere with cadherin‐mediated cell–cell junction maturation or alter integrin–ECM interactions, thereby limiting aggregate growth, although these mechanisms were not directly assessed in this study.^14^, ^23^ Similarly, too slow of a rocking speed formed exceptionally large spheroids at 7 cpm inconsistently and demonstrated less reliable urea production, possibly consistent with diffusion‐limited metabolic performance.

There is a general sinusoidal trend in the spheroid area seen over the course of the 7 days that was consistent across all speeds. The first couple days post incubation the spheroids grow in size but eventually reach an inflection point around days 3–5 at which the spheroid begins to shrink. The decrease in spheroid size is likely due to aggregate compaction associated with maturation of intercellular junctions and cytoskeletal remodeling.^23^

These data support the notion that tuning culture conditions, even in simple rocker systems, allows for scalable control of spheroid morphology without requiring substantial specialized, expensive, or high‐throughput bioreactors. These insights can be rapidly implemented in labs focused on BAL development, drug testing, and regenerative medicine for production of similar size spheroids. The scalability of hepatocyte spheroid systems has been demonstrated in literature. Suspension‐based rocker platforms enable proportional expansion through increases in culture volume while maintaining consistent cell density and mixing conditions. Large‐scale spheroid generation for bioartificial liver applications has been achieved using multi‐liter reservoirs, supporting the feasibility of producing clinically relevant spheroid numbers. In addition, prior work has shown that spheroids can be maintained with preserved viability and function under well mixed, rocked conditions with appropriate oxygenation identified as a key determinant of system performance.^26^, ^27^

Evaluation of efficiency of spheroid formation relative to number of initially seeded cells reveals that over the first few days the majority of hepatocytes are being incorporated into spheroids. Based on volumetric analysis of spheroids on day 4, there is incorporation of 70%–95% of seeded hepatocytes contributing to the total volume. It must be noted that in some of the Mayo dishes there is occasionally some sticking of hepatocytes to the sides of the dishes resulting in the 70% range in our analysis. Secondly, as discussed prior, as spheroids mature, they condense via the increased expression of intercellular adhesion molecules and therefore analysis based on diameter extrapolating to volume may underestimate the true percent incorporation of seeded hepatocytes into the spheroids. With the aforementioned caveats highlighted above, the intended observation of 86% median incorporation of hepatocytes into spheroid is to be interpreted simply as a large majority of hepatocytes are indeed incorporated and that the rocking system when optimized is reasonably efficient.

Although an exhaustive comprehensive functional profiling was not the primary objective of this study, inclusion of urea and albumin production confirms that size modulation via rocker speed is biologically meaningful. The observed peak in metabolic activity at intermediate rocking speeds is consistent with established relationships between spheroid size, oxygen diffusion, and hepatocyte function. Excessively large aggregates formed at very low speeds may be prone to diffusion limitations, whereas smaller spheroids generated at high speeds may experience shear‐related stress or insufficient cell–cell signaling. Also it is apparent that spheroid function improves over the course of the formation of the spheroid with low urea and albumin production at day 1 post followed by consistently increasing levels of production at days 7 and 10. There is likely to be a point at which the production starts to decrease; however, we did not follow the spheroid out further than day 10 for assays.

We intentionally limited functional testing to a representative metabolic readout, as detailed functional characterization is highly dependent on species, cell source, and intended downstream application. The primary aim of this work is to establish a scalable and reproducible framework for spheroid size control, upon which application‐specific functional optimization can be performed in future studies. Future studies should evaluate further functional outputs (e.g., CYP450 activity) of spheroids formed at different sizes and under varying dynamic conditions depending on the cell line of interest.

To contextualize functional performance, albumin production in spheroid cultures was compared with reported values from static 2D hepatocyte cultures. Prior studies have demonstrated albumin secretion of approximately 1.5–2.5 μg per million cells per day at day 3 and 1–2 μg per million cells per day at day 7 in 2D systems.^28^ Using an approximate conversion of 6 pg. DNA per hepatocyte^29^, our measured albumin production of 1165 ng/μg DNA/day at day 4 corresponds to ~7.0 μg per million cells per day, while 1522 ng/μg DNA/day at day 7 corresponds to ~9.1 μg per million cells per day. These values suggest higher albumin output in the spheroid configuration compared with static 2D cultures at comparable time points.

Similarly, while direct comparison of the literature is difficult due to differences in normalization and date of urea measure, we can see that the urea production of spheroids is higher than that of hepatocytes. Hepatocytes produce around 10 μg urea/μg DNA when converting some of the numbers in literature,^30^ our data suggests the spheroids produce more urea. The urea production was fairly similar to that of hepatocytes in the early days of culture while they are in the process of forming spheroids, however the amount of urea quickly surpasses that of the hepatocytes; by day 7 (when scaled to a 24 h period) was over 15 μg urea/μg DNA. These comparisons should be interpreted with caution, as the conversion from DNA to cell number assumes a diploid DNA content (~6 pg per cell). However, rat hepatocytes are known to exhibit polyploidy.^29^

## 5 LIMITATIONS

This study has several limitations. First, all experiments were conducted using primary rat hepatocytes, and extrapolation to human hepatocytes or other species should be made with caution. Significant interspecies differences exist in hepatocyte biology, including variations in cytochrome P450 isoform expression, metabolic capacity, bile acid synthesis, and cell–cell interaction dynamics.^31^, ^32^ These differences may affect spheroid formation, stability, and function, thereby limiting the direct applicability of our findings to human systems. While the exact size and rate of change in size of spheroids are likely to differ between species and cell lines, the general principle and proposed mechanisms described in this manuscript are favorable for generalizability across species. Future studies should therefore incorporate human‐derived cell sources, such as human bipotent progenitor cell line (HepaRG cells), induced pluripotent stem cell (hiPSC)‐derived hepatocytes, or primary human hepatocytes, which more closely recapitulate human liver physiology and drug metabolism.

Second, spheroid size was quantified using two‐dimensional area measurements under the assumption of ellipsoid morphology. While this approach is widely used and the large volume of samples should decrease such error associated with this assumption, irregular or non‐spherical aggregates could introduce some measurement bias. Validation of size determinations with three‐dimensional volumetric imaging or confocal reconstruction in a subset of samples would strengthen accuracy and should be considered in future work. Long‐term culture effects, including spheroid viability and structural integrity beyond 7 days, were not evaluated but could be of interest to the scientific community.

A potential limitation of rocker‐based spheroid formation is the dependence of operational parameters, such as cpm, on instrument‐specific characteristics (e.g., tilt angle and motion profile). To address this, we evaluated two independent rocker platforms and found that, after modest adjustment matching cpm, tilt angle, and inoculation density, comparable spheroid size distributions could be achieved. These findings suggest that spheroid size is primarily governed by tunable culture parameters rather than a specific device. Accordingly, while absolute parameter values may not be directly transferable across platforms, the relationship between rocking conditions and spheroid size remains consistent. Laboratories adopting this method may therefore need to perform minor calibration to achieve desired spheroid dimensions. Importantly, this reflects the adaptability of the system rather than a lack of reproducibility.

Variability in spheroid size is an important consideration for this technique. Compared with static methods such as ultra‐low attachment or microwell‐based systems, which can achieve relatively narrow size distributions, the rocker‐based method produces a broader range of spheroid sizes. For example, prior studies using microwell or tapered culture systems have reported diameter standard deviations on the order of ±10–20 μm.^13^ In contrast, the present system demonstrates greater variability, reflecting the dynamic aggregation environment. However, despite this broader distribution, we show that spheroid size can be reproducibly modulated through adjustments in rocking speed and inoculation density, providing a practical level of control for scalable suspension‐based applications. The coefficients of variation range from 18% to 47% depending on the speed and day. Day 1 post incubation has the widest variation as expected; however, by day 7, for all speeds measured, the coefficient of variation is between 21% and 24% (Figure S1) which is within the acceptable range for spheroid applications including bioartificial liver.^33^

## 6 CONCLUSION

Increasing rocker speed reduces spheroid size, while higher inoculation density increases it. This study presents a reproducible and scalable method to fine‐tune hepatic spheroid dimensions by adjusting two easily controllable parameters: rocker speed and cell inoculation density. Inclusion of focused functional assessments demonstrated that spheroid size modulation by rocker speed is biologically meaningful, with maximal urea and albumin production normalized to DNA content observed at intermediate rocking speeds. Such standardization is critical for improving the consistency of 3D hepatic cultures and optimizing their application in cryopreservation, bioartificial liver systems, and hepatocyte‐based assays. Collectively, these results lay the foundation for subsequent optimization of spheroid quality and application‐specific functional performance for translational use. By establishing simple, tunable parameters that reproducibly control spheroid size without specialized equipment, this work provides a practical and scalable strategy for advancing the standardization and translational application of hepatic spheroid systems.

## AI STATEMENT

ChatGPT5.2 (OpenAI) was utilized in the generation of the graphical abstract, Grammarly was utilized for grammatical and syntax structure optimization. All content was reviewed and approved by the authors.

## AUTHOR CONTRIBUTIONS

**Bat‐Erdene Namsrai:** Writing – original draft; writing – review and editing; investigation. **Benjamin Fisher:** Investigation; writing – original draft; writing – review and editing; methodology. **Charles Y. Lee:** Writing – original draft; writing – review and editing; methodology. **Joseph Sushil Rao:** Writing – original draft; writing – review and editing. **Anudari Chimedtseren:** Writing – original draft; writing – review and editing; investigation. **Michael G. Megaly:** Conceptualization; investigation; writing – original draft; writing – review and editing; methodology; data curation; visualization; validation; formal analysis; software. **Anthony An‐Fa Dahm Chen:** Investigation; writing – review and editing; writing – original draft; methodology. **Diane Tobolt:** Conceptualization; writing – review and editing; resources; data curation; investigation; writing – original draft. **Srivasupradha Ramesh:** Writing – original draft; writing – review and editing; investigation. **Mark Clemens:** Supervision; writing – review and editing; writing – original draft; methodology. **Scott L. Nyberg:** Conceptualization; writing – original draft; writing – review and editing; methodology. **Erik B. Finger:** Conceptualization; investigation; writing – original draft; methodology; validation; writing – review and editing; project administration; formal analysis; software; supervision. **John C. Bischof:** Supervision; resources; project administration; writing – review and editing; writing – original draft.

## FUNDING INFORMATION

This work was supported by NIH grants DK117425 (EBF, JCB), DK132211 (EBF, JCB), DK131209 (EBF, JCB), and DK106667 (SLN); NSF grant EEC‐1941543 (EBF, JCB, TLP); and a gift from the Biostasis Research Institute, funded in part through contributions from LifeGift, Nevada Donor Network, LifeSource, Donor Network West, and LifeBanc.

## CONFLICT OF INTEREST STATEMENT

The authors declare no conflicts of interest.

## Supporting information


**FIGURE S1:** Line graph of the coefficient of quartile variation from day 1 to day 7 post hepatocyte isolation for speed 7,10,12,15,18. Data showing decreasing variability of size over the course of 7 days converging toward 21%–25% coefficient of variation among all speed combinations.
**FIGURE S2:** Hepatocyte volume retained in spheroid volume. Data reflects the estimated percent of original seeded hepatocyte volume that is present within spheroids at day 4 post isolation (*n* = 5). Estimates are based on volumetric calculations assuming hepatocyte diameter of 17.5 μm. ^†^Values >100% reflect modeling assumptions and do not indicate true biologic gain.

## Data Availability

The data that support the findings of this study are available from the corresponding author upon reasonable request.

## References

[1] Carpentier B , Gautier A , Legallais C . Artificial and bioartificial liver devices: present and future. Gut. 2009;58(12):1690‐1702. doi:10.1136/gut.2008.175380 19923348

[2] Woods EJ , Thirumala S , Badhe‐Buchanan SS , Clarke D , Mathew AJ . Off the shelf cellular therapeutics: factors to consider during cryopreservation and storage of human cells for clinical use. Cytotherapy. 2016;18(6):697‐711. doi:10.1016/j.jcyt.2016.03.295 27173747

[3] Andersson TB . Evolution of novel 3D culture systems for studies of human liver function and assessments of the hepatotoxicity of drugs and drug candidates. Basic Clin Pharmacol Toxicol. 2017;121(4):234‐238. doi:10.1111/bcpt.12804 28470941

[4] Bell CC , Dankers ACA , Lauschke VM , et al. Comparison of hepatic 2D sandwich cultures and 3D spheroids for long‐term toxicity applications: a multicenter study. Toxicol Sci. 2018;162(2):655‐666. doi:10.1093/toxsci/kfx289 PMC5888952 29329425

[5] Bell CC , Hendriks DFG , Moro SML , et al. Characterization of primary human hepatocyte spheroids as a model system for drug‐induced liver injury, liver function and disease. Sci Rep. 2016;6:25187. doi:10.1038/srep25187 PMC4855186 27143246

[6] Kammerer S . Three‐dimensional liver culture systems to maintain primary hepatic properties for toxicological analysis in vitro. Int J Mol Sci. 2021;22(19):10214. doi:10.3390/ijms221910214 PMC8508724 34638555

[7] Takayama K , Kawabata K , Nagamoto Y , et al. 3D spheroid culture of hESC/hiPSC‐derived hepatocyte‐like cells for drug toxicity testing. Biomaterials. 2013;34(7):1781‐1789. doi:10.1016/j.biomaterials.2012.11.029 23228427

[8] Griffith LG , Swartz MA . Capturing complex 3D tissue physiology in vitro. Nat Rev Mol Cell Biol. 2006;7(3):211‐224. doi:10.1038/nrm1858 16496023

[9] Curcio E , Salerno S , Barbieri G , De Bartolo L , Drioli E , Bader A . Mass transfer and metabolic reactions in hepatocyte spheroids cultured in rotating wall gas‐permeable membrane system. Biomaterials. 2007;28(36):5487‐5497. doi:10.1016/j.biomaterials.2007.08.033 17881050

[10] Asthana A , Kisaalita WS . Microtissue size and hypoxia in HTS with 3D cultures. Drug Discov Today. 2012;17(15–16):810‐817. doi:10.1016/j.drudis.2012.03.004 22484546

[11] Yuhas JM , Li AP , Martinez AO , Ladman AJ . A simplified method for production and growth of multicellular tumor spheroids. Cancer Res. 1977;37(10):3639‐3643. 908012

[12] Jiang HL , Kim YK , Cho KH , et al. Roles of spheroid formation of hepatocytes in liver tissue engineering. Int J Stem Cells. 2010;3(2):69‐73. doi:10.15283/ijsc.2010.3.2.69 PMC4021799 24855543

[13] Miyamoto Y , Ikeuchi M , Noguchi H , Yagi T , Hayashi S . Spheroid formation and evaluation of hepatic cells in a three‐dimensional culture device. Cell Med. 2015;8(1–2):47‐56. doi:10.3727/215517915X689056 PMC4733911 26858908

[14] Luebke‐Wheeler JL , Nedredal G , Yee L , Amiot BP , Nyberg SL . E‐cadherin protects primary hepatocyte spheroids from cell death by a caspase‐independent mechanism. Cell Transplant. 2009;18(12):1281‐1287. doi:10.3727/096368909X474258 PMC2920600 20003757

[15] Nyberg SL . Bioartificial liver system. Mayo Foundation for Medical Education and Research; 7,160,719 B2. 2007.

[16] Brophy CM , Luebke‐Wheeler JL , Amiot BP , et al. Rat hepatocyte spheroids formed by rocked technique maintain differentiated hepatocyte gene expression and function. Hepatology. 2009;49(2):578‐586. doi:10.1002/hep.22674 PMC2680349 19085959

[17] van Zijl F , Mikulits W . Hepatospheres: three dimensional cell cultures resemble physiological conditions of the liver. World J Hepatol. 2010;2(1):1‐7. doi:10.4254/wjh.v2.i1.1 PMC2998947 21160950

[18] Li Y , Chen HS , Shaheen M , et al. Cold storage of porcine hepatocyte spheroids for spheroid bioartificial liver. Xenotransplantation. 2019;26(4):e12512. doi:10.1111/xen.12512 PMC6717030 30968460

[19] Jiang X , Xu J , Gore JC . Mapping hepatocyte size in vivo using temporal diffusion spectroscopy MRI. Magn Reson Med. 2020;84(5):2671‐2683. doi:10.1002/mrm.28299 PMC7402009 32333469

[20] Gandillet A , Alexandre E , Holl V , et al. Hepatocyte ploidy in normal young rat. Comp Biochem Physiol A Mol Integr Physiol. 2003;134(3):665‐673. doi:10.1016/s1095-6433(02)00374-4 12600676

[21] Peters SJAC , Haagsman HP , van Norren K . Arginase release by primary hepatocytes and liver slices results in rapid conversion of arginine to urea in cell culture media. Toxicol In Vitro. 2008;22(4):1094‐1098. doi:10.1016/j.tiv.2008.01.016 18348903

[22] Proctor WR , Foster AJ , Vogt J , et al. Utility of spherical human liver microtissues for prediction of clinical drug‐induced liver injury. Arch Toxicol. 2017;91(8):2849‐2863. doi:10.1007/s00204-017-2002-1 PMC5515971 28612260

[23] Lin RZ , Chou LF , Chien CCM , Chang HY . Dynamic analysis of hepatoma spheroid formation: roles of E‐cadherin and beta1‐integrin. Cell Tissue Res. 2006;324(3):411‐422. doi:10.1007/s00441-005-0148-2 16489443

[24] Torizal FG , Kimura K , Horiguchi I , Sakai Y . Size‐dependent hepatic differentiation of human induced pluripotent stem cells spheroid in suspension culture. Regen Ther. 2019;12:66‐73. doi:10.1016/j.reth.2019.04.011 PMC6933468 31890768

[25] Kyffin JA , Sharma P , Leedale J , et al. Characterisation of a functional rat hepatocyte spheroid model. Toxicol Vitro Int J Publ Assoc BIBRA. 2019;55:160‐172. doi:10.1016/j.tiv.2018.12.014 PMC6361770 30578835

[26] Lillegard JB , Fisher JE , Nedredal G , et al. Normal atmospheric oxygen tension and the use of antioxidants improve hepatocyte spheroid viability and function. J Cell Physiol. 2011;226(11):2987‐2996. doi:10.1002/jcp.22651 PMC3107922 21302300

[27] Glorioso JM , Mao SA , Rodysill B , et al. Pivotal preclinical trial of the spheroid reservoir bioartificial liver. J Hepatol. 2015;63(2):388‐398. doi:10.1016/j.jhep.2015.03.021 PMC4508211 25817557

[28] Esch MB , Prot JM , Wang YI , et al. Multi‐cellular 3D human primary liver cell culture elevates metabolic activity under fluidic flow. Lab Chip. 2015;15(10):2269‐2277. doi:10.1039/c5lc00237k PMC4422761 25857666

[29] Collins JM . RNA synthesis in rat liver cells with different DNA contents. J Biol Chem. 1978;253(16):5769‐5773. 670227

[30] Boon L , Meijer AJ . Control by pH of urea synthesis in isolated rat hepatocytes. Eur J Biochem. 1988;172(2):465‐469. doi:10.1111/j.1432-1033.1988.tb13911.x 3350009

[31] Cornu R , Rougier N , Pellequer Y , et al. Interspecies differences in the cytochrome P450 activity of hepatocytes exposed to PLGA and silica nanoparticles: an in vitro and in vivo investigation. Nanoscale. 2018;10(11):5171‐5181. doi:10.1039/c8nr00226f 29492498

[32] Takahashi S , Fukami T , Masuo Y , et al. Cyp2c70 is responsible for the species difference in bile acid metabolism between mice and humans. J Lipid Res. 2016;57(12):2130‐2137. doi:10.1194/jlr.M071183 PMC5321228 27638959

[33] Li Y , Wu Q , Wang Y , et al. Novel spheroid reservoir bioartificial liver improves survival of nonhuman primates in a toxin‐induced model of acute liver failure. Theranostics. 2018;8(20):5562‐5574. doi:10.7150/thno.26540 PMC6276288 30555564

