# Supplementary material for: The effect of rocking speed and cell inoculation density on hepatic spheroid size: A simple method for size modulation
Source: Bioeng Transl Med. 2026 Jun 5;11(5):e70154. doi: 10.1002/btm2.70154 (PMC13636245; doi:10.1002/btm2.70154)
Supplement: Supplementary file 1 [file BTM2-11-e70154-s001.docx]

Supplemental Figure 1

**Supplemental Figure 1:** *Line graph of the coefficient of quartile variation from day 1 to day 7 post hepatocyte isolation for speed 7,10,12,15,18*. Data showing decreasing variability of size over the course of 7 days converging toward 21-25% coefficient of variation among all speed combinations.

Supplemental Figure 2

**Supplemental Figure 2:** *Hepatocyte volume retained in spheroid volume.* Data reflects the estimated percent of original seeded hepatocyte volume that is present within spheroids at day 4 post isolation (n=5). Estimates are based on volumetric calculations assuming hepatocyte diameter of 17.5 um.

♱ Values > 100% reflect modeling assumptions and do not indicate true biologic gain.
